# The expression of microRNA-375 in plasma and tissue is matched in human colorectal cancer

**DOI:** 10.1186/1471-2407-14-714

**Published:** 2014-09-25

**Authors:** Lingling Xu, Minzhe Li, Min Wang, Dong Yan, Guosheng Feng, Guangyu An

**Affiliations:** Department of Oncology, Beijing Chao-Yang Hospital, Capital Medical University, Beijing, 100020 China; Department of Surgery, Beijing Chao-Yang Hospital, Capital Medical University, Beijing, 100020 China

**Keywords:** Colorectal cancer, MicroRNA, Plasma, Tissue, Biomarker, Diagnosis

## Abstract

**Background:**

MicroRNAs (miRNAs) offer great potential as cancer biomarkers. The importance of miRNAs profiling in tissue and body fluids in colorectal cancer (CRC) have been addressed respectively in many studies. The purpose of our study is to systematically assess the expression of miRNAs in cancer tissue and matched plasma samples and to evaluate their usefulness as minimally invasive diagnostic biomarkers for the detection of CRC.

**Methods:**

The study was divided into two phases: firstly, qRT-PCR based TaqMan Low Density MiRNA Arrays (TLDAs) was used to screen the differentially expressed miRNAs in 6 plasma samples of CRC patients and 6 healthy controls. Secondly, marker validation by stem-loop reverse transcription real-time PCR using an independent set of paired cancer tissues (n = 88) and matched plasma samples (CRC, n = 88; control, n = 40). Correlation analysis was determined by Pearson’s test. Receiver operating characteristic curve analyses were applied to obtain diagnostic utility of the differentially expressed miRNAs. Target gene prediction and signal pathway analyses were used to predict the function of miRNAs.

**Results:**

TLDAs identified 42 miRNAs, which were differentially expressed in patients and healthy individuals. Five of them (miR-375, miR-150, miR-206, miR-125b and miR-126*) were chosen to be validated in plasma and tissue samples. The results indicated that for plasma sample, miR-375 (*p* < 0.0001) and miR-206 (*p* = 0.0002) were dysregulated and could discriminate CRC patients from healthy controls. For tissue samples, miR-375 (*p* < 0.0001), miR-150 (*p* < 0.0001), miR-125b (*p* = 0.0065) and miR-126*(*p* = 0.0009) were down-regulated. miR-375 was significantly down-regulated and positively correlated in both tissue and plasma samples (r = 0.4663, *p* = 0.0007). Gene ontology and signal pathway analyses showed that most of the target genes that were regulated by miR-375 were involved in some critical pathways in the development and progression of cancer.

**Conclusions:**

Our results indicate that the down-regulation of miR-375 in plasma and tissue is matched in CRC. Moreover, bioinformatics prediction revealed miR-375 association with some critical signal pathways in the development and progression of CRC. Therefore, plasma miR-375 holds great promise to be an alternative tissue biomarker for CRC detection.

**Electronic supplementary material:**

The online version of this article (doi:10.1186/1471-2407-14-714) contains supplementary material, which is available to authorized users.

## Background

Colorectal cancer (CRC) is the third most common cancer and the third leading cause of cancer-related death worldwide [1]. Among Asian populations, incidence rate of CRC appeared to increase with the progressive westernization of lifestyles [2]. While advances in diagnosis and treatment have improved patient outcomes [3], long-term survival and prognosis of patients largely depend on the stage of the tumor at the time of detection. The outcomes of patients diagnosed with advanced stage disease remain quite poor [4]. Notably, most cases are diagnosed at late stages as current CRC screening tests are inconvenient and population screening rates are low. Although colonoscopy has significant utility in the detection of neoplastic lesions, its invasive nature, resulting in abdominal pain and high cost, has hampered worldwide application of this procedure [5]. Fecal-based analysis, such as occult blood immunochemical test, is convenient and inexpensive, but has low sensitivity and specificity, which impedes its utility [6]. Therefore, there is an imperative need for other minimally invasive biomarkers to complement and improve current diagnostic and prognostic tools in CRC.

MicroRNAs (miRNAs) are small, non-coding single-strand RNAs, 18–25 nucleotides in length. They are endogenously expressed and post-transcriptionally regulate gene expression by binding to 3′ untranslated region (3′UTR) of target mRNAs [7]. There is increasing evidence that miRNAs can function as tumor suppressor genes as well as oncogenes [8]. Therefore, they are important in the regulation of many biological processes, such as cell cycle, proliferation, differentiation and apoptosis [9].

There is increasing evidence that miRNAs are widely dysregulated in CRC and may have potential application for cancer diagnosis, prognosis and treatment [10–12]. For example, a recent study revealed that miR-126 was down-regulated in CRC tissue and was associated with poor survival [13]. Vickers MM et al. reported that a signature of miR-21, miR-135a, miR-335, miR-206, and let-7a was associated with stage and metastasis [14]. Among miRNAs, miR-143, miR-145, miR-21 and miR-31 are the most consistently reported to have dysregulated expression in CRC [15–17]. While miR-143 and miR-145 function as tumor suppressor genes, miR-21 and miR-31 are reported to be oncogenes.

Recently, the stability of cell-free miRNAs in body fluids enables circulating miRNAs to be potential biomarkers for noninvasive diagnosis and prognosis of CRC. Ng et al. evaluated a panel of 95 miRNAs using real-time PCR-based array and showed that plasma miR-17-3p and miR-92 were significantly elevated in CRC cases compared to controls [18]. Zantto S et al. identified that plasma levels of miR-378 could be used to distinguish CRC patients from healthy individuals [19]. However, whether dysregulated expression of miRNAs in tissue or circulation is consistent is still unknown.

The objective of our study was to correlate the differential expression of miRNAs in tissue and plasma, which could potentially serve as diagnostic biomarkers in CRC. Our results indicated that the expression of miR-375 was correlated with both tissue and plasma samples. Moreover, bioinformatics prediction revealed miR-375 association with some critical signal pathways in the development and progression of CRC. Therefore, plasma miR-375 is a potential minimally invasive biomarker for the early detection of CRC.

## Methods

This study was approved by the Clinical Research Ethics Committee of Beijing Chao-Yang Hospital. Informed consent was obtained for each patient. The clinical data were prospectively collected for all the participants involved.

### Patients and samples

A total of 140 participants were enrolled from January 2009 to December 2013. Patients used in this study had a newly diagnosed CRC before receiving any treatment. A total of 94 blood samples and a subset of 88 matched cancer tissues with adjacent normal mucosa were collected from primary CRC patients. Pathological analysis was used to confirm the histology and the patients were staged according to the tumor-node-metastasis (TNM) staging system of the International Union Against Cancer. In the control group, 46 blood samples were collected from individuals who had previously been diagnosed without any type of malignancy or other benign disease. They were matched to the CRC patients according to age and gender.

### Sample preparation and RNA isolation

Blood samples for miRNA detection were collected in EDTA-K2 tubes and processed within 1 h of collection. Blood samples were centrifuged at 1200 g for 10 min at 4°C to spin down the blood cells, and the supernatants were transferred into microcentrifuge tubes, followed by a second centrifugation at 12000 g for 10 min at 4°C. The supernatants were transferred to RNase-free tubes and stored at -80°C. The tumor and paired adjacent normal mucosa were obtained after surgical resection and immediately placed in liquid nitrogen. All analyzed tissues were homogenized before isolation. Total RNA was isolated from tissue and plasma using mirVana miRNA isolation kit (Ambion, Austin, Texas, USA) according to the manufacturer’s instructions. Briefly, 400 μl plasma and 100 mg tissue sample were used to extract total RNA. Each sample was eluted in 40 μl of RNase-free water by using Eppendorf Concentrator Plus 5301 (Eppendorf, Germany). Concentration and purification of RNA were determined spectrophotometrically by measuring its optical density (A260/280 > 2.0, A260/230 > 1.8) using NanoDrop ND-2000 Spectrophotometer (Thermo Scientific Wilmington, DE, USA).

### TaqMan microRNA array screening phase

Plasma samples of six patients diagnosed with CRC and six healthy controls were used for screening analyses. The miRNA expression profiles were performed using highly standardized qRT-PCR based TaqMan Low Density MicroRNA Arrays (TLDAs). A set of two cards (TaqMan^R^Array Human MicroRNA Card Set v2.0; Applied Biosystems, Foster City, CA, USA) enabling quantification of 754 human miRNAs and 1 endogenous controls for data normalization was used. Two sets of megaplex miRNA RT primers with special stem-loop structure allowed synthesis of all cDNAs in two separate reactions. This was carried out in accordance with the manufacturer’s instructions.

### Reverse transcription real-time PCR assay validation phase

Five miRNAs were chosen for validation based on the significance of the difference (fold change, *p*-value), previous observations and biological plausibility (according to putative miRNA targets and/or Pubmed hits when particular miRNA is combined with keyword “cancer”), and favorable expression levels (C_t_ < 30).

Validation phase was performed on a cohort of 88 CRC patients, including their plasma and tissue samples. Meanwhile, 40 healthy individual plasma samples were used as controls. cDNA was synthesized using gene-specific primers according to the TaqMan microRNA Assay protocol (Applied Biosystems). This was carried out in accordance with the manufacturer’s instructions.

Real-time PCR was performed using the Applied Biosystems 7500 Sequence Detection System. The 20 μl PCR reaction mixture included 8 μl of nuclease free water, 1 μl of PreAmp or RT product, 10 μl of 2 × Taqman (AmpErase NO UNG) Universal PCR Master Mix and 1 μl of primer and probe mix of the TaqMan MicroRNA Assay kit (Applied Biosystems). Reaction were incubated in a 96-well optical plate at 95°C for 10 min, followed by 40 cycles at 95°C for 15 s and 60°C for 1 min.

### miRNA target gene prediction, gene ontology and signal pathway analysis

The selected miRNAs were further analyzed to identify the target gene and the function. miRNA target genes were predicted by an integrated database including PicTar (http://pictar.mdc-berlin.de/), TargetScans Human 6.2 (http://www.targetscan.org/), Tarbase (http://diana.cslab.ece.ntua.gr/tarbase/) and miRecords (http://mirecords.biolead.org/).

We used the database for annotation, visualize and integrated discovery (DAVID) v6.7 (http://david.abcc.ncifcrf.gov/) to annotate the molecular function of the miRNA target genes. DIANA-mirPath (http://diana.imis.athena-innovation.gr/DianaTools/index.php?r=site/index) and Kyoto Encyclopedia of Genes and Genomes (KEGG) (http://www.genome.jp/kegg/) were used to investigate the miRNA target genes and analyze their involvement in various signal pathways.

### Statistical methods

The C_t_ value (C_t_) was calculated by SDS 2.0.5 software (Applied Biosystems) using the automatic threshold setting. All real-time PCR reactions were run in triplicates, and average threshold cycles were calculated. The average expression levels of all analyzed miRNAs were normalized using U6 as a reference gene and subsequently the 2^-Δct^ method was applied. The 2^-ΔΔct^ method was used to express the level of miRNAs in CRC tissues and matched normal mucosa samples. In the screening cohort, median values for each miRNA from the same replicates were calculated and subjected to quantile normalization to normalize the data across different arrays [20]. The normalized data were analyzed using *t*-test analysis with *p* value computations done asymptotically at *p* < 0.05. In the validation cohort, statistical differences of miRNAs levels were evaluated by the two–tailed non-parametric Wilcoxon test for 88 paired samples in tumor and adjacent normal mucosa while by the two–tailed non-parametric Mann–Whitney *U* test in plasma samples. Furthermore, spearman correlation was used to analyze the correlation between the plasma and the tissue sample. Receiver operator characteristic (ROC) analysis was applied to obtain diagnostic utility of miRNAs. Statistical analysis was performed using SPSS version 16.0 software. The *p*-values lower than 0.05 were considered statistically significant. All the graphs were performed using Graphpad prism 6 software.

## Results

### Demographics of the study

A total of 94 CRC patients and 46 healthy controls enrolled in this study. No significant differences were observed between the CRC patients and controls in the distribution of age and gender. Clinicopathological characteristics of all participants are summarized in Table 1. All the CRC cases in this study were adenocarcinomas.

**Table 1:** **Baseline characteristics of patients by miRNAs assessment set**

Characteristics	Screening set			Validation set		
	Patient (n = 6)	Control (n = 6)	p	Patient (n = 88)	Control (n = 40)	p
Average age	64.7 ± 10.9	65.8 ± 7.1	0.930	65.1 ± 11.7	65.8 ± 12.2	0.821
Gender						
Male	4	3	0.558	50	19	0.327
Female	2	3		38	21	
TNM staging						
I				7		
II	3				32	
III	3				49	
pT category						
pT 1					1	
pT 2					8	
pT 3					49	
pT 4	6				30	
Lymph nodes						
Negative	3				39	
Positive	3				49	
Vascular invasion						
Negative	3				52	
Positive	3				36	
Perineural invasion						
Negative	3				57	
Positive	3				31	
Localization						
Colon	3				52	
Rectum	3				36	
Grading						
(adenocarcinoma)						
	Low	1			14	
Moderate	4			63		
High	1				11	
Tumor diameter						
≤5 cm	2				39	
>5 cm	4				49	

### Circulating miRNA microarray profiling

To identify miRNAs that are differentially expressed in the plasma, we analyzed expression profiles of 754 miRNAs in plasma samples of six patients and six healthy controls. In the condition of *p* < 0.05 and FDR < 0.05, we observed 42 miRNAs differentially expressed between the cancer group versus the control group: 20 miRNAs were up-regulated and 22 miRNAs were down-regulated in the plasma of CRC patients. Hierarchical clustering analyze of the plasma array was shown in Additional file 1: Figure S1. In the condition of fold change > 2.0 and *p* < 0.05, we gained a set of 16 miRNAs that were differentially expressed between the CRC patients and the healthy controls (Table 2).

**Table 2:** **circulation miRNAexpression level in the screening set**

microRNA	FC	***p***
miR-342-3p	2.253333	0.04
let-7b	2.612903	0.03
miR-150	2.066667	0.01
miR-125b	2.102941	0.02
miR-375	2.162162	0.04
miR-206	2.115789	0.03
miR-127	-2.41262	0.01
miR-409-3p	-2.50883	0.03
let-7d	-2.12583	0.04
miR-520c-3p	-2.14449	0.01
miR-126	-2.65	0.01
miR-24	-5	0.01
miR-483-5p	-2.428571	0.01
miR-146a	-3.57143	0.04
mir-126*	-2.5	0.04
miR-378	-2.0819	0.04

FC: fold change (2^-ΔΔCT^,ΔCT = CT _mean_ (miRNA)-CT_mean_ (U6), ΔΔCT = ΔCT_CRC_-ΔCT_control_.

positive number refers to up-regulation; negative number refers to down-regulation of miRNA expression).

*p*: Student’s *t*-test.

### Validation of selected miRNAs by qRT-PCR

The five miRNAs which appeared to have the most potential as biomarkers were miR-375, miR-150, miR-125b, miR-206 and miR-126*. The plots of 5 miRNAs in the screening phase are in Additional file 2: Figure S2. Due to the small sample size (CRC n = 6, healthy controls n = 6) and the heterogeneity of the tumors, real-time PCR was used to validate the miRNAs.

In the validation phase, 88 paired samples of cancer tissue with adjacent normal mucosa and matched plasma samples were independently collected and 40 plasma samples of healthy individual were taken as controls. U6 was chosen as the endogenous control in data normalization and its expression was found to be stable and reproducible.

**Figure 1:**
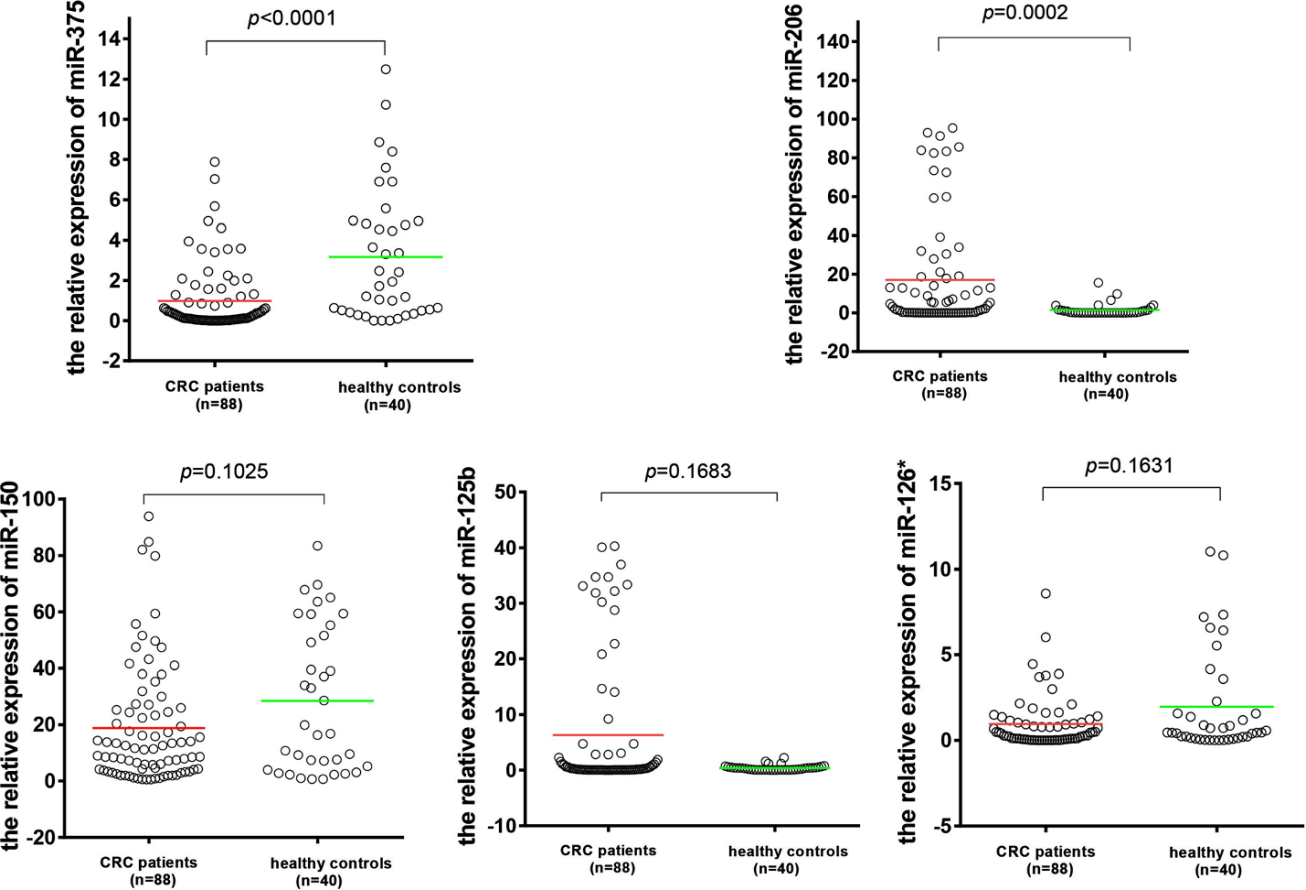
**The relative expression difference of miRNAs in plasma samples (88 CRC and 40 controls).** A single spot was the relative expression value of miRNAs of an individual patient. Lines in the middle were the mean expression value.

**Figure 2:**
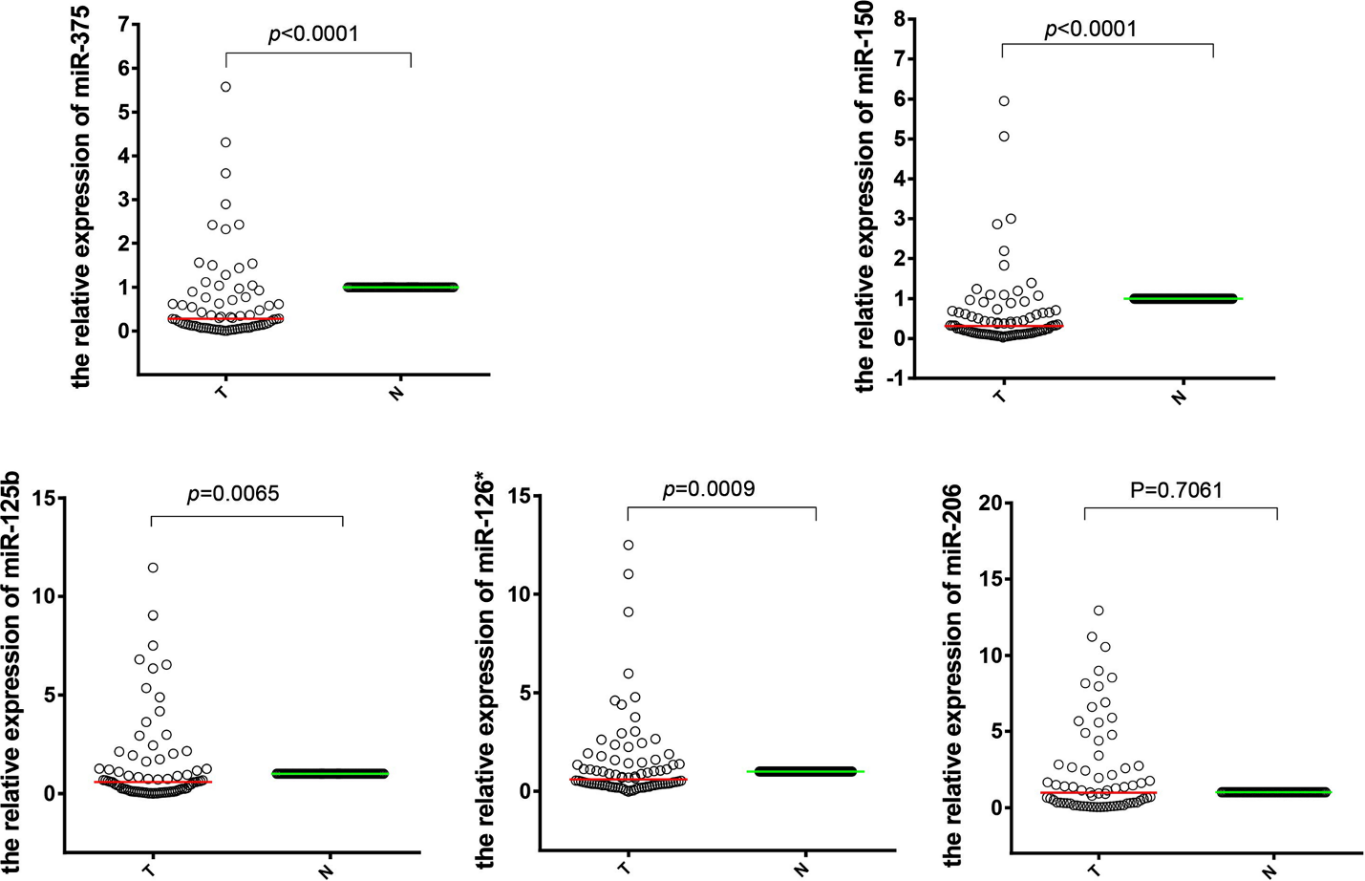
**The relative expression difference of miRNAs in tissue samples (88 cancer tissue and 88 adjacent normal mucosa).** A single spot was the relative expression value of miRNAs of an individual patient. Lines in the middle were the mean expression value.

A comparison between plasma samples of CRC patients and those of healthy controls revealed significant differences in the expression levels of miR-375 (p < 0.0001) and miR-206 (*p* = 0.0002) (Figure 1). A similar comparison of the paired cancer tissue and adjacent normal mucosa samples showed significant differences in the expression of 4 miRNAs (miR-375: *p* < 0.0001; miR-150: *p* < 0.0001; miR-125b: *p* = 0.0065; miR-126*: *p* = 0.0009) (Figure 2). However, no significant difference was observed in the levels of miR-150 (*p* = 0.1025), miR-125b (*p* = 0.1683), miR-126* (*p* = 0.1631) in plasma samples and miR-206 (*p* = 0.7061) in tissue samples. Only miR-375 was significantly down-regulated in both plasma and tissue samples.

We then conducted correlation analyses between tissue and plasma RT-PCR data while controlling for age, gender and TNM staging. The expression levels of miR-375 in tissue and plasma showed significant positive correlation (r = 0.4663, *p* = 0.0007), while miR-150, miR-125b, miR-126* and miR-206 revealed weak correlation (Table 3). The clinicopathological features of CRC patients in the validation cohort and summary of results in validation phase of the study are shown in Additional file 3: Table S1-S2. The results reveal that none of the miRNAs either in tissue or plasma samples had significant impact on clinicopathological features.

**Table 3:** **miRNA expression level in the validation set**

	Plasma (CRC n = 88 Control n = 40)		Tissue (Tumor n = 88 Normal n = 88)		Correlation	
MicroRNA	FC	***p*** ^1^	FC	***p*** ^2^	r	***p*** ^3^
miR-150	-1.5108	0.1025	-2.6244	<0.0001	0.3572	0.0175
miR-125b	1.7557	0.1683	-1.4779	0.0065	0.2211	0.0039
miR-375	-3.2146	<0.0001	-2.4130	<0.0001	0.4663	0.0007
miR-126*	-2.0565	0.1631	-1.7287	0.0009	0.3144	0.0153
miR-206	11.9144	0.0002	-1.5518	0.7061	0.2091	0.0123

CRC: Colorectal cancer.

FC: fold change (positive number refers to up-regulation; negative number refers to down-regulation of miRNA expression).

^1^: Mann–Whitney *U* test.

^2^: Wilcoxon test.

^3^:spearman correlation.

### Diagnostic value of the differentially expressed miRNA in CRC

**Figure 3:**
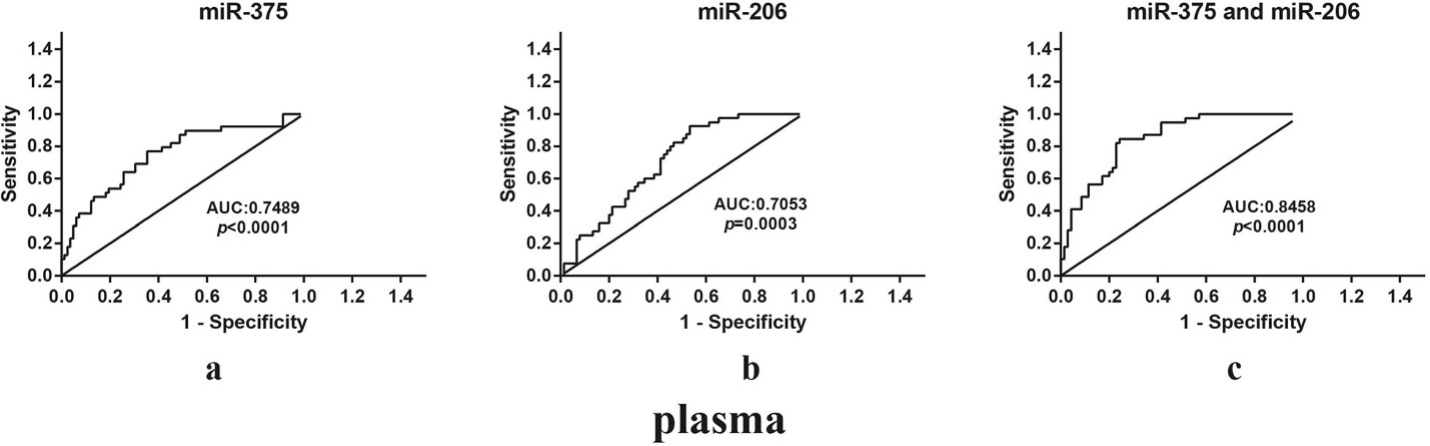
**ROC curve analysis using plasma miRNAs profile for discriminating CRC samples (88 CRC and 40 controls). (a)** miR-375 yielded areas under curve (AUC) of 0.7489 (95% CI: 0.6536-0.8442; *p* < 0.0001). **(b)** miR-206 yielded AUC of 0.7053 (95% CI: 0.6122-0.7985; *p* = 0.0003). **(c)** signature consisting of these two miRNAs yielded elevated AUC of 0.8458 (95% CI: 0.7746-0.9170; *p* < 0.0001).

**Figure 4:**
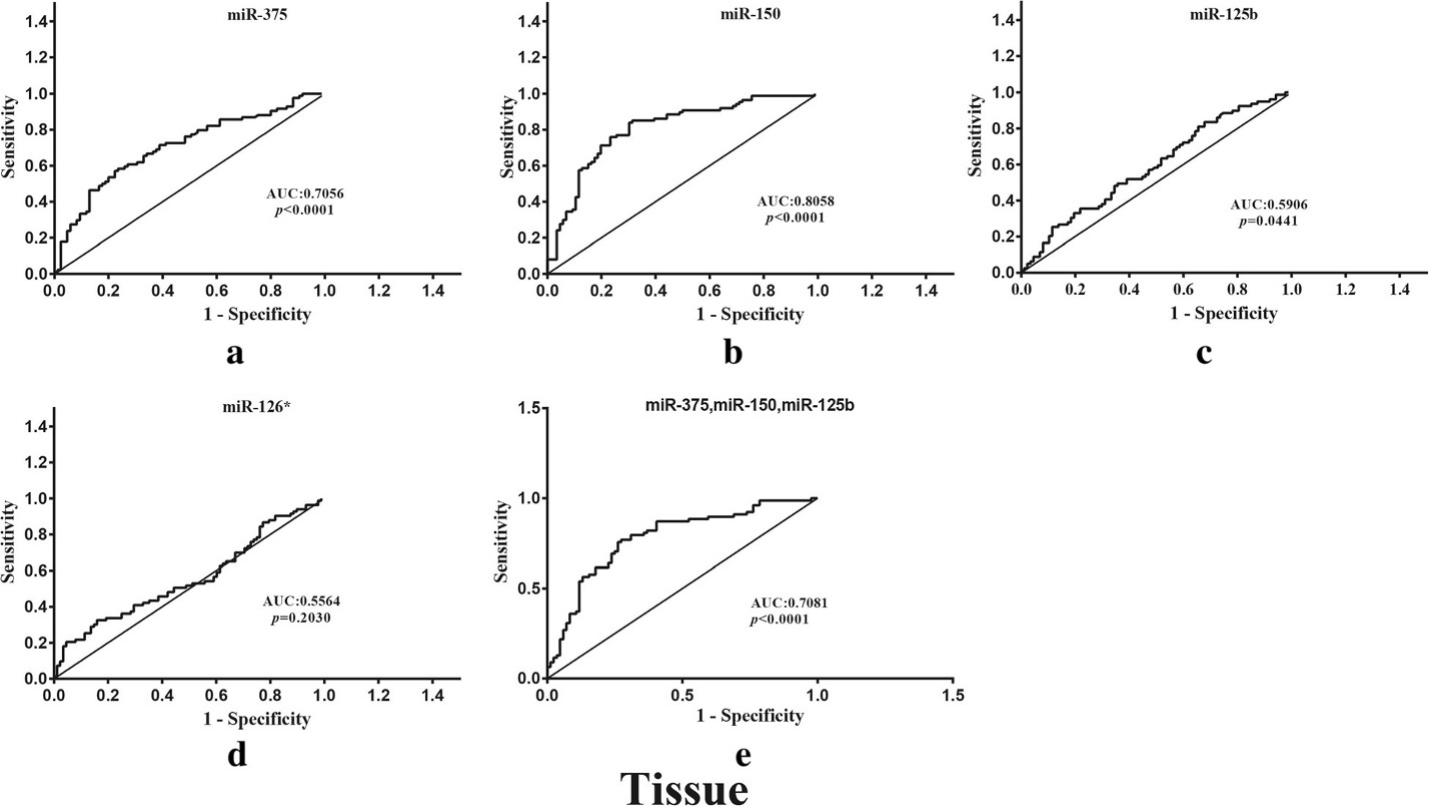
**ROC curve analysis using tissue miRNAs profile for discriminating CRC samples (88 cancer tissue and 88 adjacent normal mucosa). (a)** miR-375 yielded areas under curve (AUC) of 0.7056 (95% CI: 0.6270-0.7842, *p* < 0.0001). **(b)** miR-150 yielded AUC of 0.8058 (95% CI: 0.7396-0.8720; *p* < 0.0001). **(c)** miR-125b yielded AUC of 0.5906 (95% CI: 0.5043-0.6769; *p* = 0.0441). **(d)** miR-126* was not significant. **(e)** signature consisting of these three miRNAs yielded elevated AUC of 0.7081 (95% CI: 0.7078-0.8523; *p* < 0.0001).

To verify the diagnostic value of the miRNA signature identified in the previous cohort, the ROC curve was analyzed in the plasma and tissue respectively. In the plasma samples, the expression levels of either miR-375, miR-206 or the combination of the 2 miRNAs were useful and robust biomarkers for differentiating CRC patients from healthy controls. Area under the curve (AUC) was 0.7489 (95% CI: 0.6536-0.8442; *p* < 0.0001) for miR-375, 0.7053 (95% CI: 0.6122-0.7985; *p* = 0.0003) for miR-206 and 0.8458 (95% CI: 0.7746-0.9170; *p* < 0.0001) for the 2 markers together (Figure 3). Importantly, at the cutoff value of 0.4852 for miR-375, sensitivity was 76.92% and specificity was 64.63%. In the tissue samples, the expression levels of either miR-375, miR-150, miR-125b or the combination of the 3 miRNAs were useful biomarkers for differentiating cancer tissue from adjacent normal mucosa, with the area under the curve of 0.7081 (95% CI: 0.7078-0.8523; *p* < 0.0001) for the 3 markers together (Figure 4). At the cutoff value of 0.6071 for the 3 miRNA signatures, sensitivity was 76.92% and specificity was 72.62%. MiR-126* was not significant. Moreover, plasma miR-375 has a stronger differentiation power than tissue miR-375 individual or combination with other miRNAs. Altogether our results suggest that plasma miR-375, whose expression is correlated with tissue samples, could serve as a minimally invasive biomarker for CRC detection.

### Target prediction and function analyses of miR-375

**Figure 5:**
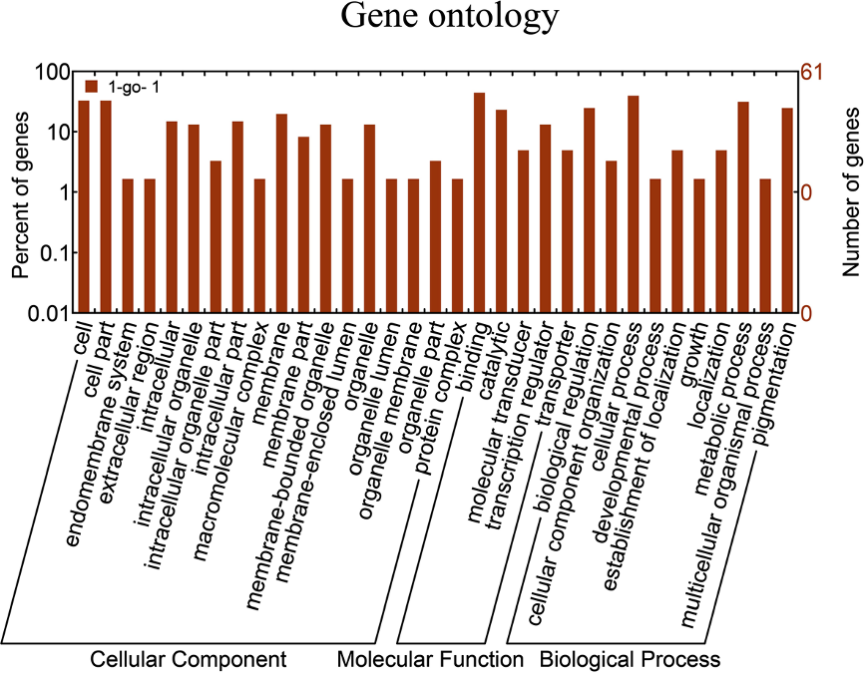
**The gene ontology (GO) analysis of the target genes of miR-375.** These genes were classified according to the gene ontology. A single bar was the number of gene in one annotation.

**Figure 6:**
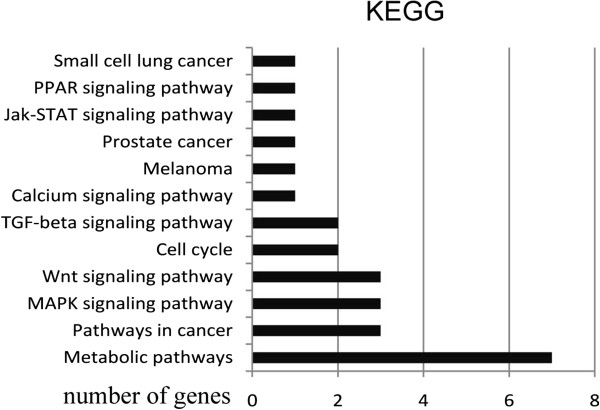
**The signal pathway analyses of the target genes of miR-375.** These genes were classified according to their function predicted by Kyoto Encyclopedia of Genes and Genomes (KEGG). A single bar was the number of gene in one pathway.

In order to investigate the role of the miR-375 in the process of CRC development and progression, we utilized four databases to select plausible targets of miR-375. To obtain reliable prediction, we extracted the target gene shared by at least 2 of these 4 databases and finally obtained a total of 69 target genes for further analysis. Then gene ontology analysis was performed using DAVID v6.7. The results showed that gene regulated by miR-375 participated in most of the important biological process such as growth or developmental process and function as transcription regulators or molecular transducers which were closely related with the development and progression of cancer (Figure 5). Some target genes such as TCF12、KLF4、ELK4 were transcription factors, whose dysregulation could induce the alteration of some significant biological processes in the cell. Signal pathway analyses showed that most of the target genes that were regulated by miR-375 were involved in some critical pathways in the development and progression of CRC, such as MAPK, Wnt, TGF-beta signal pathways (Figure 6). For example, in CRC, 90% of all tumors have a mutation in a key regulatory factor of the canonical Wnt/β-catenin signaling pathway. Wnt ligand initiates signaling through Frizzled (FZD) receptor, which was the predicted target of miR-375 [21].

## Discussion

The search for minimally invasive tools for the diagnosis of cancer has long been a goal of cancer research and has led to great interest in the field of circulation nucleic acids in plasma and serum. Since the discovery of miRNA in the circulation of cancer patients, there has been a steady increase in the study of circulating miRNAs as stable, minimally invasive biomarkers. Taqman microRNA Array was used for miRNA profiling and identified a panel of circulating miRNAs which could be minimally invasive biomarkers for CRC detection [22]. However, the question of whether circulating miRNAs can reflect the miRNAs detected in tissue remains unanswered. Our study aimed to determine whether levels of plasma miRNAs reflect those in the tissue. Therefore, our study systematically assessed the expression of miRNAs in CRC tissue and matched plasma samples.

We screened 5 miRNAs (miR-150, miR-375, miR-125b, miR-206 and miR-126*) which appeared to have the most potential as biomarkers. miR-150 is associated with survival and response to adjuvant chemotherapy [23]. But the mechanisms of the dysregulated miR-150 in CRC have not been elaborated. It is also associated with prognosis in other carcinoma, such as pancreatic,esophageal squamous cancer, lung cancer and breast cancer by targeting MUC4, ZEB1, SRCIN1 and P2X7 [24–27]. miR-125b is located at chromosome 11q23-24, a cancer-associated genomic region, which is most frequently involved in breast and lung cancer [28, 29]. It is also down-regulated in CRC tissue and associated with tumor progression, invasion and poor prognosis [30, 31]. The target of miR-125b is Mcl-1,Bcl-w,IL-6R. To our best knowledge, there are few studies on miR-206 in CRC. A study revealed that miR-206 was down-regulated in CRC tissue samples and was associated with clinical stage, lymph node metastasis and poor survival [14]. However, the mechanisms of miR-206 in CRC remain largely unknown. A recent study of miR-206 in melanoma showed that it targeted CDK4, Cyclin C and Cyclin D1 which were cell cycle genes. Therefore, miR-206 induced G1 arrest and acted as a tumor suppressor in melanoma [32]. Studies on miR-126* in CRC are few. miR-126* is the complementary sequence of miR-126. However, the expression of miR-126 has been validated in CRC and shown to be down-regulated in CRC tissues that expressed high levels of CXCR4. The low miR-126 and high CXCR4 protein expression was associated with distant metastasis, clinical TNM stage and poor survival [13]. miR-126 overexpression inhibits cell proliferation, migration and invasion and induced cell arrest in the G0/G1 phase of CRC cells. The results revealed that miR-126 function as a tumor suppressor in CRC cells by regulating CXCR4 expression via the AKT and ERK1/2 signaling pathways [33]. For miR-375, in vitro and animal studies showed that pancreatic miRNA-375 directly targets PDK1, plays key roles in glucose regulation of insulin gene expression and β-cell growth and is down-regulated in pancreatic carcinoma [34, 35]. Recently, several studies have indicated that miR-375 expression is frequently down-regulated in colorectal cancer tissue compared to the non-tumor counterparts and could be used as new biomarkers for CRC [36, 37]. MiR-375 inhibits colorectal cancer growth by targeting PI3K/Akt signaling pathway [38]. Another study revealed that miR-375 reduced cell viability through the induction of apoptotic death by targeting YAP1 [39]. Such observations only suggested the role of miRNA in tissue or plasma samples alone.

Of the 5 miRNAs investigated in our study, only miR-375 showed consistent correlations between tissue and plasma samples. The expression of miR-150, miR-125b, miR-126* and miR-206 were dysregulated in CRC, which was corresponding to the previous studies but their correlation between tissue samples and plasma samples were weak. Moreover, plasma miR-375 with a sensitivity of 76.92%, specificity of 64.63% and AUC of 0.7489 has a stronger differentiation power than tissue miR-375 individually or in combination with other miRNAs. To investigate possible involvement of miR-375 in CRC, we applied gene ontology and KEGG analysis and found that miR-375 target a large number of genes involved in some critical signaling pathways in cancer and served as transcriptional regulator in cancer significant signal pathways [40]. To our best knowledge, our study is the first one to evaluate the expression of miR-375 in CRC tissue and matched plasma samples. The results suggest that plasma miR-375, whose expression is consistent between tissue samples and plasma samples, could serve as a minimally invasive biomarker for CRC detection. MiR-375 appears to provide us a way to detect disease by using easily available clinical specimens. However, there were no significant correlations between the expression level of miRNAs in plasma or tissue samples and the clinicopathological features.

Unexpectedly, while miR-206 and miR-125b were down-regulated in tissue samples, they were up-regulated in plasma samples. The search for a possible explanation revealed that miR-206 is a circulating muscle-specific miRNA. The expression of serum miR-206 is significantly higher in rhabdomyosarcoma [41] and in the early stage of 4-(methylnitrosamino)-1-(3-pyridyl)-1-butanone (NNK) induced lung carcinogenesis [42]. However, the expression of miR-206 is down-regulated in some tumor tissue samples, such as breast, gastric and colorectal cancer [14, 43, 44]. Presently, few reports have been published on circulating miR-206 in CRC. In contrast, miR-125b is multifaceted, with the ability to function as a tumor suppressor or an oncogene, depending on the cellular context. There is no report about the expression of miR-125b in plasma and matched tissue samples in CRC. Recently, a study revealed that the expression level of miR-125b in exosomes were significantly lower in melanoma patients compared with disease-free patients with melanoma and healthy controls [45]. Circulating miRNAs packaged in exosomes can be delivered to recipient cells where they exert gene silencing through the same mechanism as cellular miRNAs [46]. Exosomes can provide a suitable material to measure circulating miRNAs in melanoma. The expression of miR-125b has not been consistent so far and the reason of inconsistent expression pattern of miR-206 and miR-125b in tissue and fluid samples remains largely unknown.

Some studies found the same trend of alteration between circulating miRNAs and tissue miRNAs. For instance, miR-375 and miR-141 were both highly expressed in serum and tissue samples of prostate cancer patients [47]. However, Wulfken et al. found that 109 miRNAs were at higher levels in renal cell carcinoma patients’ serum, but only 36 miRNAs were up-regulated in the corresponding tissue samples. It is possible that only a subset of circulating miRNAs have tumor-specific origins [48]. These data suggest that cells have a mechanism in place to select specific miRNAs for cellular release or retention [49].

Some limitations need to be taken into account when interpreting the results of this study. First, the sample size is small, especially in the marker screening phase. Second, the amount of some miRNAs in plasma are too low to be accurately quantified, therefore, some potential relevant markers could not be considered. Third, in our study, the target genes and the function of miRNAs were predicted by an integrated database. Out of the numerous databases available to predict the target gene, we chose four databases, namely PicTar, Targetscan, Tarbase and miRecords. This is because some studies have revealed that PicTar and Targetscan have high specificities and are more accurate in predicting the target genes [50], while Tarbase and miRecords included some target genes which had been validated in the research [51]. Further functional assays of miR-375 need to be done to elucidate the role of circulating miRNAs in CRC.

## Conclusions

Circulating miRNAs appear to be potentially useful biomarkers for cancer detection. Identifying the relationship between circulating miRNAs and tissue miRNAs will be helpful in understanding the useful of circulating miRNAs. Plasma miR-375 is matched with tissue sample and has the potential to be an alternative of tissue biomarker. Our study serves as an exploratory basis for further investigation of the tissue and plasma miRNAs in larger sample size. Further research on a multi-marker circulating based test might be a promising approach to identify the asymptomatic individuals with colorectal cancer prior to more invasive examination.

## Electronic supplementary material

Additional file 1: Figure S1: The hierarchical clustering analyze of the plasma array. The cluster analysis of 42 differential miRNAs was performed by Cluster 3.0 software. Red represents up-regulation and green represents down-regulation. (JPEG 304 KB)

Additional file 2: Figure S2: The relative expression difference of miRNAs in plasma samples in the screening phase (6 CRC and 6 healthy controls). A single spot was the relative expression value of miRNAs of an individual patient. Lines in the middle were the mean expression value. (JPEG 290 KB)

Additional file 3: Table S1: The relationship between the expression of plasma miRNAs and the clinicopathological features in the validation cohort. Each miRNAs were expressed by median values (25%percentile-75%percentile). The p-values lower than 0.05 were considered statistically significant. **Table S2.** The relationship between the expression of tissue miRNAs and the clinicopathological features in the validation cohort. Each miRNAs were expressed by median values (25%percentile-75%percentile). The p-values lower than 0.05 were considered statistically significant. (DOCX 23 KB)

## Abbreviations

MiRNA: MicroRNA
TLDAs: TaqMan Low Density MiRNA Arrays
CRC: Colorectal cancer
3′UTR: 3′ untranslated region
GO: Gene ontology
KEGG: Kyoto Encyclopedia of Genes and Genomes
TNM: Tumor-node-metastasis
DAVID: The database for annotation, visualize and integrated discovery
FZD: Frizzled.
