# Supplementary figures and images for: The expression of microRNA-375 in plasma and tissue is matched in human colorectal cancer
Source: BMC Cancer. 2014 Sep 25;14:714. doi: 10.1186/1471-2407-14-714 (PMC4181388; doi:10.1186/1471-2407-14-714)

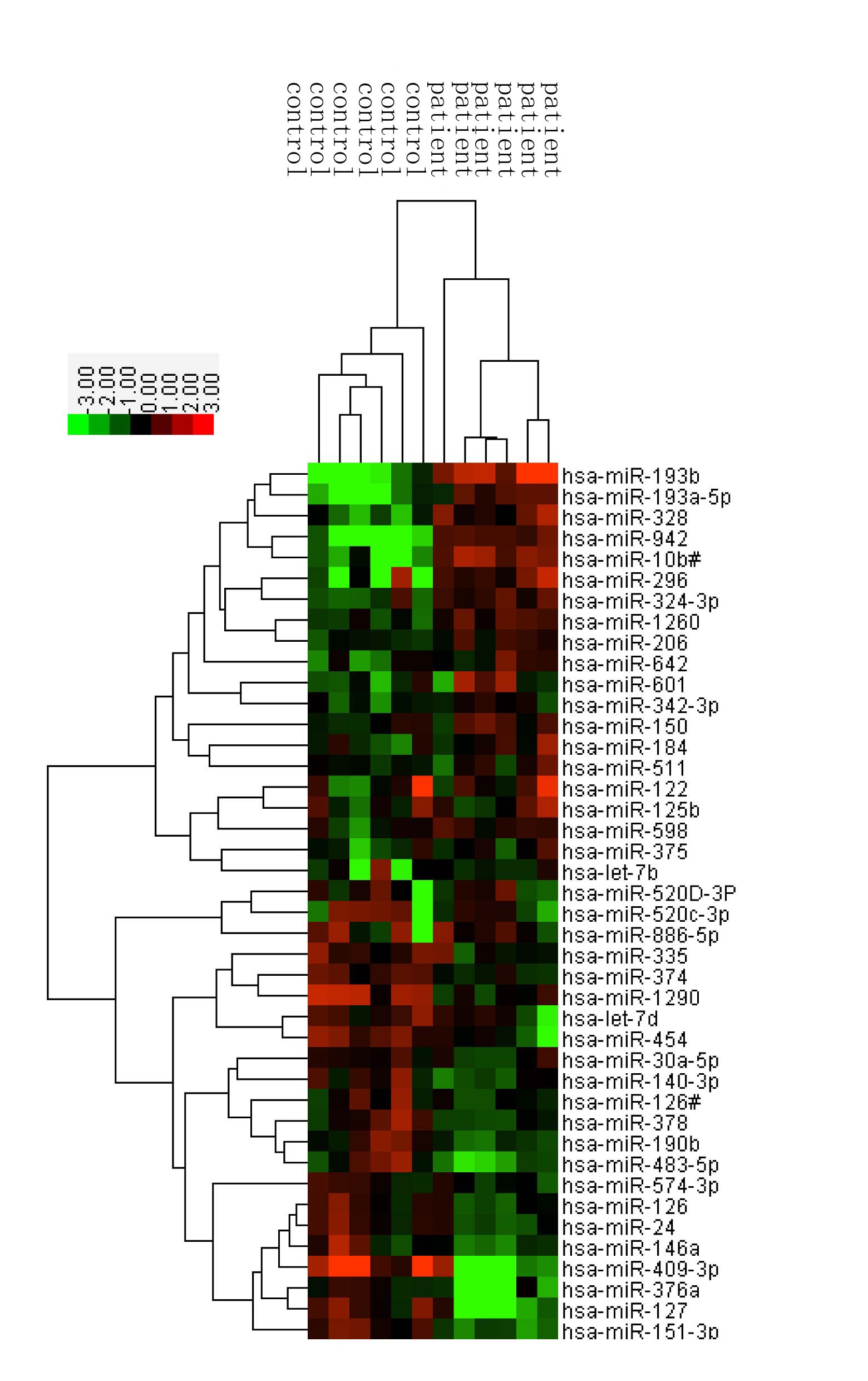

Supplement: Supplementary file 1 — Additional file 1: Figure S1: The hierarchical clustering analyze of the plasma array. The cluster analysis of 42 differential miRNAs was performed by Cluster 3.0 software. Red represents up-regulation and green represents down-regulation. (JPEG 304 KB) [file 12885_2014_4880_MOESM1_ESM.jpeg]

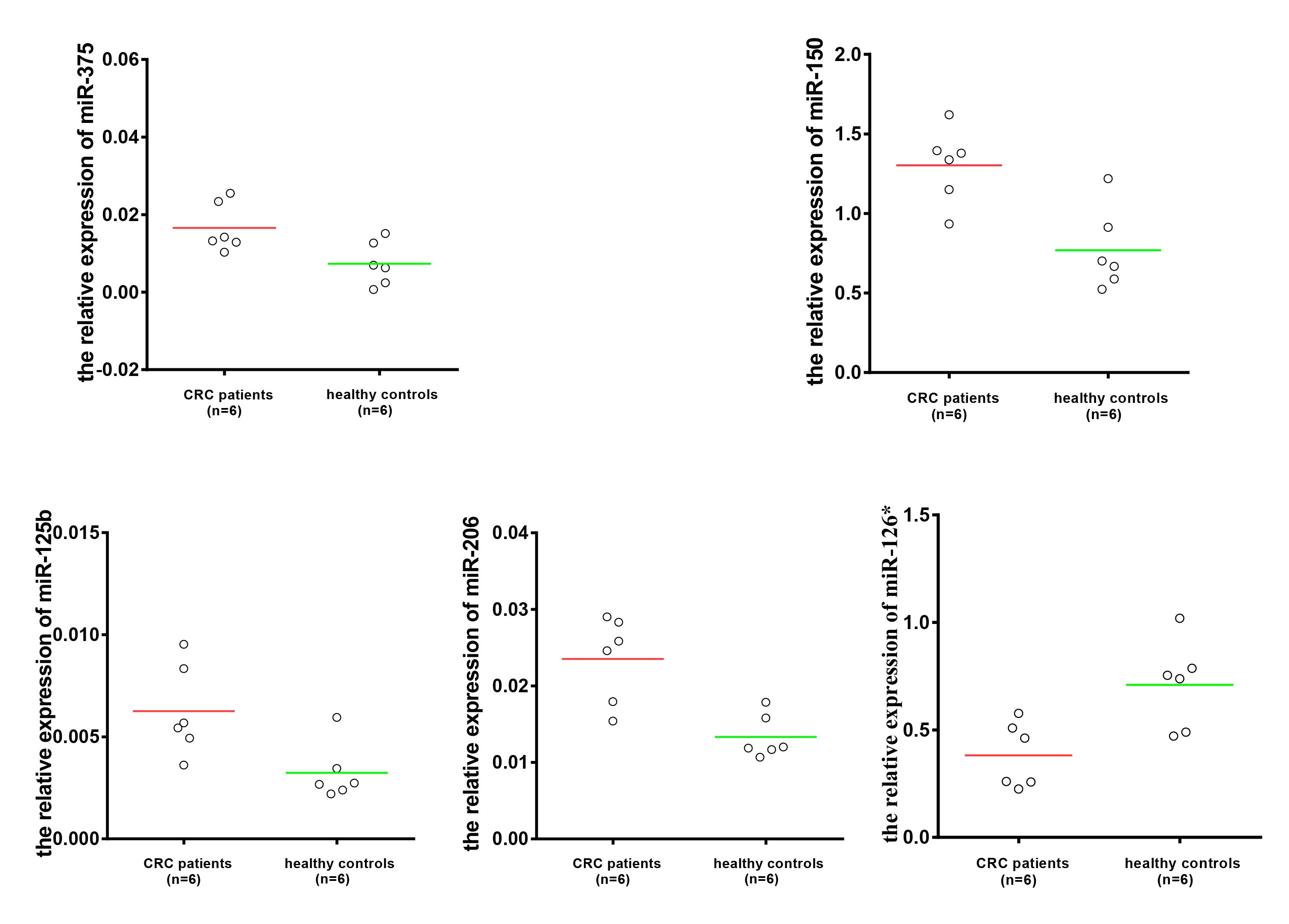

Supplement: Supplementary file 2 — Additional file 2: Figure S2: The relative expression difference of miRNAs in plasma samples in the screening phase (6 CRC and 6 healthy controls). A single spot was the relative expression value of miRNAs of an individual patient. Lines in the middle were the mean expression value. (JPEG 290 KB) [file 12885_2014_4880_MOESM2_ESM.jpeg]
