# Supplementary material for: The expression of microRNA-375 in plasma and tissue is matched in human colorectal cancer
Source: BMC Cancer. 2014 Sep 25;14:714. doi: 10.1186/1471-2407-14-714 (PMC4181388; doi:10.1186/1471-2407-14-714)
Supplement: Supplementary file 3 — Additional file 3: Table S1: The relationship between the expression of plasma miRNAs and the clinicopathological features in the validation cohort. Each miRNAs were expressed by median values (25%percentile-75%percentile). The p-values lower than 0.05 were considered statistically significant. Table S2. The relationship between the expression of tissue miRNAs and the clinicopathological features in the validation cohort. Each miRNAs were expressed by median values (25%percentile-75%percentile). The p-values lower than 0.05 were considered statistically significant. (DOCX 23 KB) [file 12885_2014_4880_MOESM3_ESM.docx]

| S Table 1 The relationship between the expression of plasma miRNAs and the clinicopathological features in the validation cohort | | | | | | |
| --- | --- | --- | --- | --- | --- | --- |
|  | n | miR-375 | miR-206 | miR-125b | miR-126* | miR-150 |
| TNM staging |  |  |  |  |  |  |
| I+ II | 39 | 0.25（0.05-1.20） | 1.71（0.06-11.86） | 0.16(0.02-2.67) | 0.22(0.06-0.81) | 8.41（2.92-24.49） |
| III | 49 | 0.36（0.04-2.27） | 1.36（0.07-18.64） | 0.31(0.03-4.73) | 0.72(0.07-1.74) | 13.76（6.21-36.57） |
| P value |  | 0.53 | 0.84 | 0.43 | 0.08 | 0.21 |
| pT category |  |  |  |  |  |  |
| pT 1+ pT 2 | 9 | 0.10（0.04-1.09） | 11.50（0.02-21.10） | 0.04（0.02-0.47） | 0.18(0.07-0.79) | 12.56（3.06-16.88） |
| pT 3+ pT 4 | 79 | 0.33（0.05-1.99） | 1.17（0.06-7.52） | 0.16（0.01-1.48) | 0.44(0.07-1.30) | 11.07（3.96-27.32） |
| P value |  | 0.52 | 0.61 | 0.65 | 0.50 | 0.73 |
| Lymph nodes |  |  |  |  |  |  |
| Negative | 39 | 0.25（0.05-1.20） | 1.71（0.06-11.86） | 0.16(0.02-2.67) | 0.22(0.06-0.81) | 8.41（2.92-24.49） |
| Positive | 49 | 0.36（0.04-2.27） | 1.36（0.07-18.64） | 0.31(0.03-4.73) | 0.72(0.07-1.74) | 13.76（6.21-36.57） |
| P value |  | 0.53 | 0.84 | 0.43 | 0.08 | 0.21 |
| Vascular invasion |  |  |  |  |  |  |
| Negative | 52 | 0.37（0.04-1.96） | 0.32（0.05-8.35） | 0.19(0.02-6.97) | 0.34(0.06-1.04) | 12.95（3.01-31.37） |
| Positive | 36 | 0.28（0.07-1.73） | 6.21（0.07-18.94） | 0.42(0.03-3.93) | 0.67(0.07-2.99) | 11.95（4.85-26.98） |
| P value |  | 0.78 | 0.24 | 0.84 | 0.36 | 0.98 |
| Perineural invasion |  |  |  |  |  |  |
| Negative | 57 | 0.25（0.04-1.29） | 2.22（0.07-14.06） | 0.19(0.02-4.73) | 0.27(0.07-1.27) | 8.96（4.10-27.20） |
| Positive | 31 | 0.41（0.09-3.40） | 1.64（0.07-39.29） | 0.28(0.02-2.78) | 0.70(0.04-1.26) | 13.67（2.92-41.04） |
| P value |  | 0.46 | 0.71 | 0.70 | 0.74 | 0.80 |
| Grading |  |  |  |  |  |  |
| low | 14 | 0.35（0.09-2.31） | 0.20（0.01-11.20） | 0.03(0.01-0.87) | 0.44(0.20-1.61) | 13.52（2.86-29.76） |
| moderate | 63 | 0.36（0.04-2.08） | 2.22（0.14-12.96） | 0.31(0.04-11.63) | 0.27(0.05-1.22） | 12.56（4.23-27.32） |
| high | 11 | 0.13（0.02-0.74） | 0.21（0.01-3.81） | 0.03(0.01-0.87) | 0.92（0.03-1.10） | 8.41（2.84-41.04） |
| P value |  | 0.54 | 0.17 | 0.10 | 0.73 | 0.96 |

| S Table 2 The relationship between the expression of tissue miRNAs and the clinicopathological features in the validation cohort | | | | | | |
| --- | --- | --- | --- | --- | --- | --- |
|  | n | miR-375 | miR-206 | miR-125b | miR-126* | miR-150 |
| TNM staging |  |  |  |  |  |  |
| I+ II | 39 | 0.001  (0.000-0.003) | 1.37e-005  (5.66e-006-3.95e-005) | 0.001  (8.10e-005-0.002) | 0.005（0.001-0.008） | 0.005  （0.002-0.012） |
| III | 49 | 0.001  (0.000-0.002) | 1.49e-005  (3.810e-006-4.780e-005) | 0.001  (5.420e-005-0.002) | 0.004（0.001-0.007） | 0.005（0.002-0.010） |
| P value |  | 0.397 | 0.938 | 0.942 | 0.369 | 0.892 |
| pT category |  |  |  |  |  |  |
| pT 1+ pT 2 | 9 | 0.001  (0.000-0.003) | 8.000e-006  (5.525e-006-1.660e-005) | 6.750e-005  (5.065e-005-0.001) | 0.006  （0.001-0.009） | 0.005  （0.001-0.042） |
| pT 3+ pT 4 | 79 | 0.001  (0.000-0.002) | 1.520e-005  (4.820e-006-4.220e-005) | 0.000  (9.540e-005-0.001) | 0.004（0.001-0.007） | 0.005  （0.002-0.012） |
| P value |  | 0.390 | 0.257 | 0.245 | 0.664 | 0.644 |
| Lymph nodes |  |  |  |  |  |  |
| Negative | 39 | 0.001  (0.000-0.003) | 1.37e-005(5.66e-006  -3.95e-005) | 0.001  (8.10e-005-0.003) | 0.005（0.001-0.008） | 0.005  （0.002-0.012） |
| Positive | 49 | 0.001(0.000-0.002) | 1.49e-005  (3.810e-006-4.780e-005) | 0.001  (5.420e-005-0.004) | 0.004（0.001-0.007） | 0.005（0.002-0.010） |
| P value |  | 0.397 | 0.938 | 0.942 | 0.369 | 0.892 |
| Vascular invasion |  |  |  |  |  |  |
| Negative | 52 | 0.001  (0.000-0.004) | 8.700e-006  (3.940e-006-3.890e-005) | 0.000  (9.830e-005-0.001) | 0.005（0.001-0.009） | 0.007（0.003-0.014） |
| Positive | 36 | 0.001  (0.000-0.001) | 2.200e-005  (1.300e-005-4.530e-005) | 0.000  (5.268e-005-0.001) | 0.003（0.001-0.006） | 0.004（0.002-0.007） |
| P value |  | 0.060 | 0.081 | 0.170 | 0.077 | 0.069 |
| Perineural invasion |  |  |  |  |  |  |
| Negative | 57 | 0.001（0.000-0.002） | 1.280e-005  (4.270e-006-3.375e-005) | 0.000  (6.810e-005-0.001) | 0.004（0.002-0.007） | 0.004（0.002-0.009） |
| Positive | 31 | 0.001（0.000-0.003） | 1.950e-005  (5.850e-006-6.480e-005) | 0.000  (0.000-0.001) | 0.003（0.001-0.008） | 0.007（0.003-0.014） |
| P value |  | 0.837 | 0.088 | 0.488 | 0.238 | 0.171 |
| Grading |  |  |  |  |  |  |
| low | 14 | 0.001（0.000-0.003） | 8.000e-006  (3.990e-006-3.490e-005) | 0.000  (2.708e-005-0.001) | 0.005（0.002-0.009） | 0.005（0.003-0.011） |
| moderate | 63 | 0.001（0.000-0.002） | 1.865e-005  (6.003e-006-4.058e-005) | 0.000  (8.100e-005-0.001） | 0.003  （0.001-0.007） | 0.006  （0.002-0.012） |
| high | 11 | 0.002（0.001-0.003） | 5.410e-006  (2.610e-006-5.310e-005) | 0.000  （0.000-0.001） | 0.006  （0.005-0.010） | 0.003  （0.001-0.015） |
| P value |  | 0.290 | 0.224 | 0.620 | 0.124 | 0.732 |
